# The Relationship Between Prostate Cancer Aggressiveness and Glycemic Levels in Patients Submitted to Radical Prostatectomy

**DOI:** 10.4021/wjon664e

**Published:** 2013-05-06

**Authors:** Suzana Cristina Goncalves, Rafael de Moraes Siqueira, Marcus Vinicius F Nogueira, Joao Antonio Pereira-Correia, Fernando Pires Vaz, Wilza Arantes Ferreira Peres

**Affiliations:** a Department of Nutritional Biochemistry, Faculty of Nutrition, Federal University of Rio de Janeiro, Avenida Carlos Chagas Filho 373, Ilha do Fundao, Rio de Janeiro, RJ, Brazil; b Department of Urology, Servidores do Estado Federal Hospital, Rua Sacadura Cabral 120, Saude, Rio de Janeiro, RJ, Brazil; c Department of Nutritional Biochemistry, Faculty of Nutrition, Federal University of Rio de Janeiro, Avenida Carlos Chagas Filho 373, Ilha do Fundao, Rio de Janeiro, RJ, Brazil

**Keywords:** Prostate cancer, Gleason score, Hyperglycemia, Radical prostatectomy

## Abstract

**Background:**

The relationship between hyperglycemia and prostate cancer remains controversial. According to current hypotheses, elevated serum glucose levels may lead to disease development or disease prevention. Our study examined the potential correlation between pre-operative glycemic levels of patients with prostate cancer and the grade of tumor aggressiveness.

**Method:**

We studied the case files of patients with a diagnosis of prostate cancer who had received putatively curative cancer surgery at the Urology Department of the Servidores do Estado Federal Hospital (RJ/Brazil). We transcribed information related to glycemia - collected up to 3 months before the surgery - and the histopathological grade of tumor aggressiveness (Gleason score) of the surgically removed prostates.

**Results:**

We analyzed 42 people who met the inclusion criteria. Based on Gleason scores, among the normoglycemic patients, we detected low, moderate, and highly aggressive neoplasias in 13%, 53%, and 36% of the cases, respectively. For the hyperglycemic group, these rates were 30%, 60%, and 10%, respectively. Normoglycemic patients had primary Gleason grade 3 in 40% of the cases and grade 4 in 60% of the cases. For the hyperglycemic patients, these rates were 90% and 10%, respectively (P < 0.05 vs. grade 3 group).

**Conclusion:**

Both Gleason score and primary Gleason grade were lower in hyperglycemic patients with prostate cancer than in normoglycemic patients, suggesting a “protective action” of hyperglycemic states.

## Introduction

Prostate cancer is currently considered to be a consequence of male aging. Studies based on the histopathological analysis of cadavers show that approximately 30% of men aged 40 have a latent foci for the disease and, at 80 years of age, this index reaches a value of approximately 70% [1, 2]. In Brazil, it is considered to be the third most common cause of death by neoplasias for men [3]. According to the most recent estimates from the National Cancer Institute of Brazil, an average of 70 new cases per 100,000 inhabitants was predicted in 2012 [4].

Originally described in the 1960s, the Gleason score is considered to be the most widely known and most often used tool with which to evaluate prostate cancer aggressiveness [5]. The score is based on cell differentiation and organization. The more disorganized and undifferentiated the neoplastic cells are, the greater the Gleason score and, therefore, the greater the tumor aggressiveness [5].

The sum of the value for the primary Gleason grade (the most prevalent cell organization in the prostate tissue) and the secondary grade (the second most prevalent type of cell organization observed in the prostate tissue) corresponds to the Gleason score. Gleason scores in the range of 2 to 6 correspond to a low-aggression malignant neoplasia and 7 to a moderately aggressive tumor. Scores in the range of 8 to 10 correspond to a more aggressive prostate neoplasia [6].

The specific causes determining the development and progression of the disease remain uncertain [7]. However, more and more evidence is being collected that relates the development of prostate cancer to genetic and environmental factors [7]. The latter include the consumption of saturated fat [8, 9], obesity [10-12] and alcohol consumption [13].

Recently, hyperglycemia has been associated with the development of cancer. Some studies claim that the higher risk of carcinogenesis stems from mitogenic action induced by insulin [14], which is generally found to be high in hyperglycemic patients. However, the relationship between hyperglycemia and prostate cancer remains controversial. In this neoplasia type, high glucose levels can both lead to and prevent the development of the disease, according to current hypotheses [15].

Our study evaluated whether and to what extent pre-operative levels of glycemia in patients receiving cancer surgery to remove the prostate correlate with prostate cancer aggressiveness.

## Methods

We studied the case files of male patients, aged between 50 and 80 years, with a diagnosis of prostate cancer, who were treated as outpatients and received surgery aimed at curing the cancer from the Urology Department of the Servidores do Estado Federal Hospital (SEFH) (Rio de Janeiro/RJ/Brazil). We evaluated the subjects’ data from the pre-operative period. Those who agreed to participate in the study signed an informed consent form.

We transcribed from each file the data relating to glycemia and the histopathological grade of tumor aggressiveness for prostate samples obtained through radical prostatectomy (pelvic surgery to remove the prostate). Glycemia was recorded at two different points, up to 3 months prior to the date of surgery. We also collected data related to age, place of residence, daily drug use habits, and place of origin.

We applied the following exclusion criteria: no pre-operative prostate biopsy with histopathology; oral hypoglicemiant drug intake; and fewer than two pre-operative glycemia records in the case reports. For inclusion in this study, we required: a prostate biopsy with a diagnosis of prostate cancer; urological surgery with the intention of curing the prostate cancer; and two records, with an inter-record interval of at least 7 days, of pre-operative glycemia.

We performed a descriptive population analysis and a comparative analysis of the values for the variables using the Statistical Package for the Social Sciences TM (SPSS) program, version 19.0 (IBM). We used the Kolmogorov-Smirnov test to verify the normality of the datasets. We evaluated quantitative variables with a normal distribution using Student’s t-test. For non-parametric variables, we applied the Mann-Whitney test. To correlate the numerical variables, we used Spearman or Pearson’s correlation test. We established a significance level of P < 0.05.

## Results

We analyzed 150 case reports of patients who had received radical prostatectomy for prostate cancer at SEFH’s Urology Department between January 2011 and August 2012. Of these 150 men, 42 met the inclusion criteria.

In terms of the demographic data, we observed that the patients’ average age was 65 years old, ranging from 53 to 78. Forty men (95%) were born in the city of Rio de Janeiro (RJ/Brazil), and the remaining two (5%) were from the city of Duque de Caxias (RJ/Brazil). In terms of drugs used daily, we observed that 27 patients (64%) used anti-hypertensive drugs. The others (36%) did not use any medication regularly.

Considering 99 mg/dL as the maximum value for normal glycemia, we divided the patients into three groups: normoglycemic (maximum glycemia of 99 mg/dL at two tests), hyperglycemic (glycemia above 100 mg/dL at two tests) and variable glycemic (glycemia ≤ 99 mg/dL in one test and ≥ 100 mg/dL in another tests). The prostate cancer aggressiveness data for the normoglycemic and hyperglycemic patients are presented in Table 1 and 2.

**Table 1:** Complications of a Meckel’s Diverticulum

Age (years)	Normoglycemic Patients (glycemia ≤ 99 mg/dL)				
	Pre-operative glycemia (mg/dL)		Tumor aggressiveness		
	Test 1 (mg/dL)	Test 2 (mg/dL)	Primary Gleason grade	Secondary Gleason grade	Gleason score
63	95	99	3	4	7
70	92	84	3	4	7
55	84	67	4	4	8
53	88	91	4	3	7
63	88	99	4	3	7
71	78	85	4	3	7
64	94	82	4	4	8
66	92	90	4	5	9
53	93	93	3	4	7
61	90	78	3	4	7
63	84	81	4	4	8
54	95	84	3	3	6
65	65	72	4	5	9
71	71	98	4	3	7
66	66	79	3	3	6

**Table 2:** Complications of a Meckel’s Diverticulum

Age (years)	Hyperglycemic Patients (glycemia ≥ 100 mg/dL)				
	Pre-operative glycemia (mg/dL)		Tumor aggressiveness		
	Test 1 (mg/dL)	Test 2 (mg/dL)	Primary Gleason grade	Secondary Gleason grade	Gleason score
72	126	124	3	3	6
54	100	109	3	4	7
68	409	132	3	3	6
67	103	101	4	5	9
70	119	137	3	4	7
59	136	130	3	4	7
70	138	126	3	4	7
73	100	115	3	4	7
60	112	124	3	3	6
54	100	102	3	4	7

With respect to Gleason scores, we observed that of the 15 normoglycemic patients evaluated, 2 (13%) had low-aggression prostate cancer (Gleason score 6), 8 (53%) had moderately aggressive cancer (Gleason score 7), and 5 (36%) had very aggressive cancer (Gleason score 8 or 9). In the group of 10 hyperglycemic patients, we found that 3 (30%) had low-aggression cancer (Gleason score 6), 6 (60%) had moderately aggressive cancer (Gleason score 7), and 1 (10%) had very aggressive cancer (Gleason score 9) (Table 3). All excised tumors were confined to the prostate accordingly to the 7th edition of TNM classification of malignant tumors [16].

**Table 3:** Complications of a Meckel’s Diverticulum

Tumor aggressiveness based on Gleason score	No. (percentage) of normoglycemic patients	No. (percentage) of hyperglycemic patients	Pearson Correlation Coefficient	P value
2 - 6 (not very aggressive)	2 (13%)	3 (30%)	-0.297	0.05
7 (moderately aggressive)	8 (53%)	6 (60%)		
8 - 10 (very aggressive)	5 (36%)	1 (10%)		

With respect to primary Gleason grade, six normoglycemic patients (40%) had grade 3 presentation and nine (60%) had grade 4. Primary Gleason grade 3 was detected in nine hyperglycemic patients (90%) and one (10%) had a grade 4 presentation (Table 4).

**Table 4:** Complications of a Meckel’s Diverticulum

Tumor aggressiveness based on primary Gleason grade	No. (percentage) of normoglycemic patients	No. (percentage) of hyperglycemic patients	Pearson Correlation Coefficient	P value
3 (less aggressive)	6 (40%)	9 (90%)	-0.368	0.01
4 (more aggressive)	9 (60%)	1 (10%)		

Our statistical analysis indicated that the “glycemia” variable correlated negatively with both Gleason score (Pearson’s coefficient of correlation = -0.297; P = 0.05) and primary Gleason grade (Pearson’s coefficient of correlation = -0.368; P = 0.01).

## Discussion

There are various theories correlating states of chronic hyperglycemia with the development of cancer. The increase in levels of blood insulin observed in these patients, for example, leads to a decrease in liver synthesis of type 1 insulin-like growth fac­tor binding protein (IGFBP), as well as a decrease in serum levels of IGFBP [17-19]. Hyperinsulinemia has also been associated with a reduced plasma level of type 2 IGFBP [20]. The reduced level of these binding proteins then results in an increase in the bioavailability of type 1 insulin-like growth factor (IGF). The binding of insulin and IGF1 to their respective receptors promotes cell proliferation and inhibits apoptosis in various kinds of human tissues. These effects can, in turn, contribute to carcinogenesis [20].

The liver is the main source of circulating IGF-1. Synthesis of this growth factor is stimulated by the action of the somatotropic hormone (growth hormone). Serum insulin acts on the liver, increasing the number of receptors for this organ’s growth hormone, and thus for the production of IGF-1 [21, 22].

Many cancer cells have insulin receptors [23], especially for the expression of isoform A (IR-A). IR-A activation causes more mitogenic effects than metabolic effects [18]. As a consequence of this excess of type A isoform receptors, insulin can favor cancer progression and facilitate the neoplastic growth of tumors that remain clinically irrelevant.

Nonetheless, the relationship between hyperglycemia and the development of prostate cancer remains controversial. Current theories claim that high levels of serum glucose can both cause this neoplasia’s development and prevent it [24]. Some studies propose that the chronic plasma increase in glucose found in type 2 diabetes patients functions as a protective factor since, in these patients, blood levels of androgens are routinely low [24]. The fact that androgens work to stimulate the growth of prostate neoplasias [25] could explain this preventative action. At the same time, a genetic factor may also be responsible for this proliferative effect, since the HNF-1β allele (hepatocyte nuclear factor 1 homeobox B) increases the risk of developing type 2 diabetes and also reduces the likelihood of developing prostate cancer [26]. Two large meta-analyses based on recent epidemiological studies obtained similar results. In these studies, having diabetes functioned as a protective factor for the development of the disease [27, 28].

There is also controversy about the relationship between levels of glycemia and prostate cancer aggressiveness. Some studies have shown that elevated blood levels are associated with neoplasias with a higher Gleason score and, therefore, more aggressive cancer, while others claim the opposite. A multi-centric North American study conducted by Kang and collaborators in 2012 retrospectively evaluated 15,330 diabetic and non-diabetic men who received radiation treatment for prostate cancer, which had been detected by prostate biopsy [29]. Through comparative logistic regression of the data from 92% of the non-diabetic subjects and 8% of the diabetic subjects (type I and II), they found that diabetic patients were at a greater risk of developing more aggressive tumors (Gleason score 8-10). Another study, using a similar methodology, evaluated 16,286 men and yielded very similar results when evaluating ethnicity and diabetes as risk factors for neoplastic prostate aggressiveness [30]. The authors concluded that diabetes is a factor that functions independent of ethnicity for the development of aggressive tumors.

In our study, however, we verified that for patients with prostate cancer and hyperglycemia, both the Gleason score and the primary Gleason grade value were lower than in normoglycemic patients than in hyperglycemic patients, suggesting a “protective action” for hyperglycemic states. We believe that a fundamental methodological difference may explain the difference in our results versus the previous studies mentioned. Analyses that suggest that diabetes functions as a risk factor for more aggressive tumors were performed using data from prostate biopsies, unlike our findings, which came from histopathological analyses of the surgical specimen. Various studies have already established that the analysis of prostate cancer tissue obtained in biopsies is extremely useful for diagnosing prostate cancer, but generally fails in helping to grade the disease. Rates of underestimation and overestimation can reach 40%, according to a recent meta-analysis [31].

Studies that evaluate the correlation between glycemia and histopathology data from surgical specimens are rare. Jayachandran and collaborators conducted a retrospective study involving 1,262 patients who had received radical prostatectomy, of whom 19% were diabetic. They identified more aggressive tumors in diabetic, obese, and Caucasian people relative to patients not in these groups [32]. Unlike our evaluation, however, they did not mention whether the patients had used anti-diabetic drugs, which might have influenced the results because using metformin, for instance, has already been tied to anti-oncogenesis in some neoplasias [33]. None of the subjects in our cohort used hypoglycemic drugs.

Among the limitations of our research, we should highlight the very design of the study (observational transversal), the lack of correlation between the aggressiveness of prostate cancer and other data that might influence it, such as body mass index, ethnicity, levels of glycated hemoglobin and serum testosterone, as well as the small number of patients identified with hyperglycemia, in comparison to the control group. We should emphasize, however, that this sample size, although small, should be considered significant from a proportional point of view since we determined that approximately 25% of the patients in the cohort were hyperglycemic. This figure is above the prevalence rate for diabetes mellitus in Brazil, which is at 7.4% according to the “Diabetes Census” performed in nine state capitals [34].

There are previous analyses of other variables that might influence tumor aggressiveness, which were not recorded in our study, and those that might not yet have been defined. Kim and collaborators were able to observe a correlation between Gleason score and serum levels of glycated hemoglobin in patients receiving radical prostatectomy at four Veterans’ Hospitals in the United States [35]. A retrospective analysis of data from 247 patients demonstrated that patients with elevated serum levels of glycosylated hemoglobin (> 7.8%) had more aggressive tumors, but certain methodological limitations, highlighted by the authors themselves, compromised the clinical application of the study. Similarly, correlations with body mass index [36, 38] and serum testosterone [39-41] remains inconclusive, with some studies showing a protective effect and others characterizing elevated glycemic levels as a risk factor.

The influence of the period of time of hyperglycemia on the aggressiveness of prostate cancer also needs to be examined more closely. Our analysis is based on glycemic levels collected in the period of 90 days prior to surgery, but the total time of the “hyperglycemic state” was not measured. A Swedish cohort study observed a greater risk of developing prostate neoplasia only in the first year after being diagnosed with diabetes (relative risk = 2.8; P < 0.05). After this period, the presence of diabetes became a protective factor (relative risk = 0.5; P < 0.05) [42]. Nevertheless, a study involving 13 years of tracking, known as the Cancer Prevention Study, identified the exact opposite pattern. In patients with a diagnosis of diabetes 5 years earlier, the relative risk for developing prostate cancer was 0.84, whereas for patients diagnosed more than 5 years previously, the relative risk was higher, at 1.56 [43].

Our observational study revealed evidence of the effects of glycemic changes on pre-existing prostate tumors. We strongly recommend that clinical trials with a random selection of patients, a long follow-up period and, as a result, greater scientific consistency, be performed so we can better understand the effects of glycemic dysfunction on the clinical picture of prostate cancer.

## Declaration

The work was performed in Department of Urology - Servidores do Estado Federal Hospital, Rua Sacadura Cabral, No.120, Saude, Rio de Janeiro, RJ, Brazil.
